# Cholesterol Crystal Embolism and Chronic Kidney Disease

**DOI:** 10.3390/ijms18061120

**Published:** 2017-05-24

**Authors:** Xuezhu Li, George Bayliss, Shougang Zhuang

**Affiliations:** 1 Division of Nephrology, Tongji University School of Medicine, Shanghai 200120, China; xuezhuli@gmail.com; 2 Department of Medicine, Rhode Island Hospital and Alpert Medical School, Brown University, Providence, RI 02903, USA; gbayliss@lifespan.org

**Keywords:** cholesterol crystal embolism, atheroembolic renal disease, chronic kidney disease

## Abstract

Renal disease caused by cholesterol crystal embolism (CCE) occurs when cholesterol crystals become lodged in small renal arteries after small pieces of atheromatous plaques break off from the aorta or renal arteries and shower the downstream vascular bed. CCE is a multisystemic disease but kidneys are particularly vulnerable to atheroembolic disease, which can cause an acute, subacute, or chronic decline in renal function. This life-threatening disease may be underdiagnosed and overlooked as a cause of chronic kidney disease (CKD) among patients with advanced atherosclerosis. CCE can result from vascular surgery, angiography, or administration of anticoagulants. Atheroembolic renal disease has various clinical features that resemble those found in other kidney disorders and systemic diseases. It is commonly misdiagnosed in clinic, but confirmed by characteristic renal biopsy findings. Therapeutic options are limited, and prognosis is considered to be poor. Expanding knowledge of atheroembolic renal disease due to CCE opens perspectives for recognition, diagnosis, and treatment of this cause of progressive renal insufficiency.

## 1. Introduction

Cholesterol crystal embolism (CCE) occurs when small pieces of atheromatous plaques from the aorta or other major arteries break off and shower small arteries cholesterol crystal emboli. CCE is an important type of crystallopathy, as outlined in an elegant review from Mulay and Anders summarizing its pathophysiological mechanisms [1]. As a multisystemic disease, CCE involves many organs including kidneys, skin, gastrointestinal tract, eyes, muscle, central nervous system, and extremities. Embolic events usually are iatrogenic from surgical or invasive endovascular manipulation, but can also be spontaneous. Autopsy studies reported that kidneys were involved in 74% of cases of CCE. CCE is becoming a common cause of renal insufficiency in adults over the age of 60 with advanced atherosclerosis [2,3]. The clinical presentation of CCE is diverse, with limited therapeutic options and poor long-term outcomes for resulting atheroembolic renal disease [4]. This life-threatening disease may be underestimated and overlooked as a cause of chronic kidney disease (CKD) in patients with diffuse atherosclerosis and/or undergoing interventional cardiac catheterization, and its diagnosis and management still remain unresolved. In this review, we discuss the recent advances in the clinical and pathological findings in CCE.

## 2. Etiology

Atherosclerosis is a common disorder of arteries in which plaques composed of cholesterol, fatty substance, and other cellular waste on the intimal surface of arterial vessel walls form a plaque. The fibrous cap covering the plaques appears vulnerable to rupture, exposing the soft, cholesterol core of the plaque to the circulation. Then cholesterol crystals (CCs) move into the bloodstream and get stuck in arterioles (usually in medium-sized arteries 100 to 200 μm in diameter) [4]. These CCs emboli lead to narrowing or obliteration of the arteriole lumen, resulting in ischemia and infarction of tissue distal to the cholesterol crystal emboli [5], potentially affecting the kidneys, skin, gastrointestinal system, eyes, muscles and bones, brain, nerves, visceral organs, and extremities [5]. The anatomical proximity of the kidneys to the abdominal aorta and degree of renal blood flow make the kidney a frequent target organ for cholesterol atheroembolism. Acute kidney failure is possible if acute arterial occlusion occurs when the artery that supplies blood to the kidney suddenly becomes blocked.

CCE is largely iatrogenic, often associated with aortic surgery, arterial invasive procedure with manipulation of the aorta or other major arteries, such as angiography, left heart catheterization, and coronary angioplasty. It is estimated that about 30–85% of CCE patients have a history of invasive vascular procedure in the preceding 3 months, while only 4.3% had cholesterol embolism in age-matched controls that did not have invasive vascular procedure [6,7]. CCE is also a rare complication in patients undergoing the administration of anticoagulants (including heparin, low molecular weight heparin, warfarin, dabigatran) and thrombolytic therapy [2,7,8,9,10]. The incidence of CCE induced by these agents may increase rapidly with their widespread use for artrial fibrillation and acute myocardial infarction in recent years. One proposed explanation for anticoagulant and thrombolytic agents related CCE is that anticoagulants and thrombolytic treatment agents may initiate the rupture of plaques by causing internal hemorrhage or dissolution of fibrous caps, exposing the underlying low-density lipoprotein-derived CCs to the systemic circulation. Studies showed that anticoagulation agents could be implicated in 7% of biopsy-proven CCE without arterial invasive procedures [2]. The incidence of CCE among patients taking warfarin is low (0.7% to 1.0%) [2]. Patients with diffuse atherosclerosis may also suffer spontaneous detachment of plaques or continuous low-grade migration of CCs from the aortic wall. Spontaneous CCE is rare, with reported frequency ranging from 1.9% to 13% [2,11].

## 3. Epidemiology

The exact incidence of CCE is not known, as it is often underdiagnosed [2]. The reported incidence varies from 1.1% to 4.5% due to different study designs and diagnostic criteria [2,12]. Mayo and Swartz surveyed 402 consultation charts retrospectively, suggesting that the incidence of clinically detectable atheroembolism amounted to at least 4% of all inpatients examined [13]. Fukumoto and colleagues reported that 1.4% patients, among 1786 patients who underwent left-heart catheterization, were diagnosed with CCE, and that 64% of those patients had renal damage [14]. The incidence of CCE in autopsy studies is similar to that found in renal biopsy series [2,15]. CCE accounted for 7% of all causes of acute renal injury in a study of 259 patients, age 60 or older, who underwent renal biopsy [16].

CCE is becoming a common cause of renal failure in older adults with atherosclerosis [3]. Scolari et al. observed that 60% of 354 patients diagnosed with CCE were older than 70 years [7]. The other risk factors for CCE are the same as those for atherosclerosis, including male gender, Caucasian race, tobacco use, ischemic cardiovascular disease, cerebrovascular disease, hypertension, hypercholesterolemia, diabetes, hypercoagulability, abdominal aortic aneurysm, peripheral vascular disease, and family history [2,3,4,11].

## 4. Pathogenesis

The exact mechanism underlying CCE is not fully understood. The local tissue necrosis and inflammatory reaction caused by CCs play a significant role in the pathogenesis of CCE. Furthermore, activation of renin-angiotensin-aldosterone system, complement activation also contribute to the development of CCE.

### 4.1. Necroinflammation

CCs cause tissue injury directly through mechanical obstruction that leads to vascular obstruction, tissue ischemia and cell necrosis, referred as “necroinflammation” [1]. Animal experiments showed that crystals lodged in a vessel lumen induce platelet aggregation, thrombosis and complete arterial obstruction [17]. The local inflammatory reaction caused by CCs may play a crucial role in the luminal occlusion and subsequent renal insufficiency. The CCs trigger a foreign-body inflammatory reaction around the arterioles, involving macrophage infiltration and foreign body giant cell reaction. A number of studies have described a series of reactions of the arterial wall to CCs. Jones and Iannacone divided the inflammation reaction into three phases [18]. In the early phase, fresh crystal emboli cause endothelial injury, including swelling of cells with dilated endoplasmic reticulum and osmotic damage in mitochondria [17]. Crystal emboli also cause an early histiocytic response, including transient appearance of neutrophils and eosinophils. The intermediate phase is characterized by a giant-cell infiltration and intimal proliferation. The late phase involves the encasement of the crystals by histiocytes, more intimal proliferation, and intravascular fibrosis [19].

CCs are emerging as an endogenous initiator of inflammation. Martinon, Tschopp, and colleagues found that crystals induce interleukin-1β activation in mononuclear phagocytes through the NLRP3 inflammasome [20]. The NLRP3 inflammasome is an intracellular platform that translates various danger signals into the activation of caspase-1 and the secretion of interleukin-1β. Duewell and colleagues [21] confirmed that CCs also activate the NLRP3 inflammasome to trigger the secretion of mature interleukin (IL)-1β and α from macrophages, leading to cell necrosis. In addition, Corr et al. report that CCs induce IL-α/β production through the activation of Syk and PI3K in human macrophages and dendritic cells [22]. Moreover, a recent study shows that CCs directly bind to the human macrophage-inducible C-type lectin (hMincle) and induce pro-inflammatory cytokine, such as tumor necrosis factor(TNF)and macrophage inflammatory protein 2(MIP-2), release [23].

### 4.2. Activation of Renin-Angiotensin-Aldosterone System (RAAS)

Blood pressure in patients with CCE is usually hard to control, with some evidence suggesting that CCE contributes to activation of RAAS. Tanaka et al. reported a case of severe hyperreninemic hypertension associated with spontaneous renal CCE [24]. Obstruction of arcuate arteries, interlobular arteries, afferent arterioles, or glomerular capillaries by CCs reduced focal blood perfusion and induced activation of RAAS, which exerts deleterious effects through oxidative stress leading to apoptosis, inflammation, and fibrosis [25]. In addition, RAAS also can promote vascular and tissue remodeling, further contributing to chronic kidney disease. Clinically, RAAS inhibitors may have a potential benefit for improvement of renal outcomes in CCE.

### 4.3. Complement Activation

Complement factors are involved in the inflammatory response associated with atherosclerosis. CCs activate both classical and alternative complement pathways [26,27]. In experiments with cultured endothelial cells, CCs activated complement factors, specifically C5a, leading to leukocyte aggregation and endothelial damage by release of leukocyte enzymes and oxidants [28]. A recent study demonstrated that CCs activated the classical complement pathway and TNF release, which caused endothelial activation in vitro [29]. Furthermore, CCs induced soluble terminal C5b-9 formation and C3c deposition on the CCs surface, increased the expression of complement receptor 3(CD11b/CD18) on monocytes and granulocytes in human whole blood, and reconstituted high density lipoprotein (rHDL) attenuated the complement activation induced by CCs [27]. Therefore, CC activation of complement is an important mechanism controlling the release of proinflammatory cytokines and inflammatory responses.

## 5. Pathology

**Figure 1:**
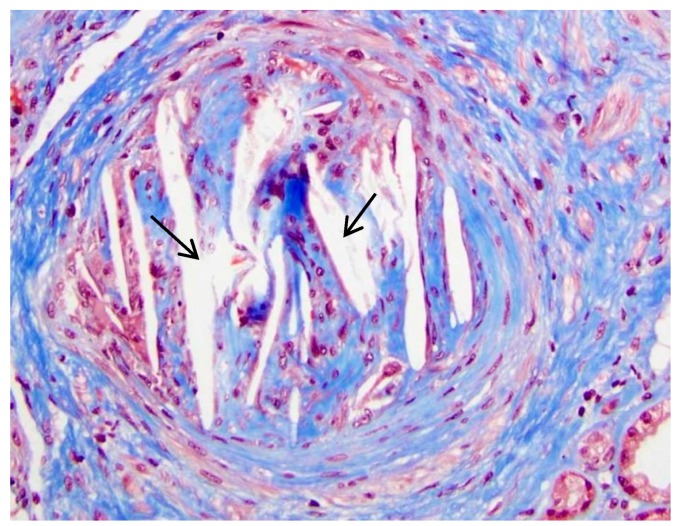
Pathological changes of cholesterol crystal embolism. Renal biopsy from a 68-year-old man with peripheral vascular disease, hypertension, abdominal aortic aneurysm shows the arcuate artery occluded by an organizing atheroembolus, consisting of cholesterol clefts (arrows), macrophages and lymphocytes, and fibroblasts. The patient had acute renal failure with serum creatinine = 11.8 mg/dL. Masson Trichrome, Original Magnification 200×. Image courtesy of Isaac E. Stillman, M.D., Beth Israel Deaconess Medical Center/Harvard Medical School.

Renal complications of CCE are usually difficult to estimate clinically, owing to the syndrome’s propensity to mimic other renal disorders. Kidney biopsy is considered the definitive test for diagnosis of CCE. On renal biopsy, the characteristic lesion of CCE is occlusion of cholesterol emboli in the lumina of arcuate, interlobular arteries, and glomeruli. The emboli of CCs generally are defined by the empty, biconvex, and needle-shaped clefts, appearing as “ghosts”, because CCs usually dissolve during routine histologic preparation procedures. However, in frozen sections, the crystals are birefringent under polarized light and give positive histochemical reactions for lipids [30]. Interlobular and arcuate arteries showed a perivascular polymorphonuclear and eosinophilic infiltration. In the later stage of the disease, perivascular fibrosis occurs around the occluded vessels. Glomeruli can have normal morphology in the initial stage, but ischemic retraction of podocyte foot processes, focal segmental glomerulosclerosis (FSGS), interstitial fibrosis, and tubular atrophy can be seen frequently due to ongoing ischemic injury in the later stages of the disease (Figure 1). Generally, immunofluorescence staining for immunoglobulin A (IgA), IgG, IgM, C3, C1q, fibrin, kappa, and lambda light chains are negative [31]. Renal biopsy has a sensitivity of about 75%. Involvement of renal vasculature is patchy, and the diagnosis may be missed if not enough sections are examined [32].

Renal biopsy is often not performed due to older age, comorbidity, and prior CKD, leading to a delay in diagnosis. Skin, muscle, or gastrointestinal (GI) biopsies may also provide evidence of cholesterol clefts in tissue and indirectly confirm the diagnosis without a renal biopsy. Skin biopsy is favored as an alternative to renal biopsy because it is less invasive, and skin lesions are involved in nearly 90% of CCE [30].

## 6. Clinic Presentations

### 6.1. Renal Complication of Cholesterol Crystal Embolism (CCE)

Renal complications of CCE, also known as atheroembolic renal disease (AERD), take two forms: acute/subacute and chronic (Table 1). The acute/subacute form presents as a sudden decline in renal function resulting from a massive shower of cholesterol crystal emboli due to abrupt rupture of unstable plaques. Acute CCE occurs within seven days after an inciting event with multiorgan CCE involvement. In the subacute presentation, renal insufficiency arises weeks or months after the inciting event. It can be explained by recurrent cholesterol crystal emboli showers and the foreign-body reaction.

A chronic subset of CCE, presenting as a slowly progressive form, is likely due to frequent and slow crystal migration from ruptured atherosclerotic lesions [7,33,34,35]. It can be clinically silent when small non-significant cholesterol crystal embolism occurs in kidneys. Most published series emphasize the acute form of AERD. However, CCE is often overlooked because it mimics symptoms of several other chronic kidney diseases, such as chronic glomerular sclerosis, systemic vasculitis, ischemic nephropathy, hypertensive nephrosclerosis. Diagnosis of chronic CCE is difficult, often made postmortem. Interestingly, Greenberg and colleagues evaluated clinical presentations and pathological findings of 24 patients with CCE. Nine patients had nephrotic range proteinuria. Light microscopy and electron microscopy study disclosed focal segmental glomerulosclerosis (FSGS), largely of the cellular variant FSGS with podocyte hypertrophy and capillary loop collapse. It should be recognized that FSGS presenting with heavy proteinuria can occur as part of a chronic disorder, and CCE should be considered in the differential diagnosis of secondary FSGS [36].

There is no specific laboratory test for AERD [37]. Blood tests for elevation of serum creatinine can confirm the diagnosis of acute renal injury or chronic kidney disease but do not identify cause. Microscopic hematuria and minimal proteinuria is typically found on urinalysis. Nephrotic range proteinuria (>3 g/day) can be seen on occasion. Eosinophilia is common in CCE patients; the incidence varies from 14% to 71%. Eosinophilia is usually transient, due to release of interleukin-5 by activated T cells [32]. Urinary eosinophilia has also been reported in cases [38]. Elevated erythrocyte sedimentation rate, C-reactive protein levels, and transient C3 hypocomplementemia may be present, but not specific.

Renal pathology can also include membranous nephropathy [36] and purpura nephritis [39]. CCE was associated with renal dysfunction caused by CC-induced ischemic interstitial damage (tubular atrophy and fibrosis) in those scenarios. CCE may also occur even in end-stage renal disease patients who are undergoing maintenance dialysis [4,40,41].

Cholesterol embolism can also occur in transplanted kidney. CCE in renal allografts is a rare condition with a reported incidence of about 0.39% to 0.47% [42]. Common clinical manifestation, risk factors, and potential triggers are the same as cholesterol crystal embolism in native kidneys. Atheroemboli may arise from either donor or recipient vessels. Embolization of donor origin occurred early after transplantation, causing primary renal allograft failure, while cholesterol embolization from recipient causes a late chronic allograft dysfunction, which usually occurs years after transplantation in a stable graft [43,44]. CCE is an important and under-reported cause of renal allograft dysfunction and should be considered when either primary or late renal graft failure occurs [45].

### 6.2. Extra-Renal Manifestation

**Figure 2:**
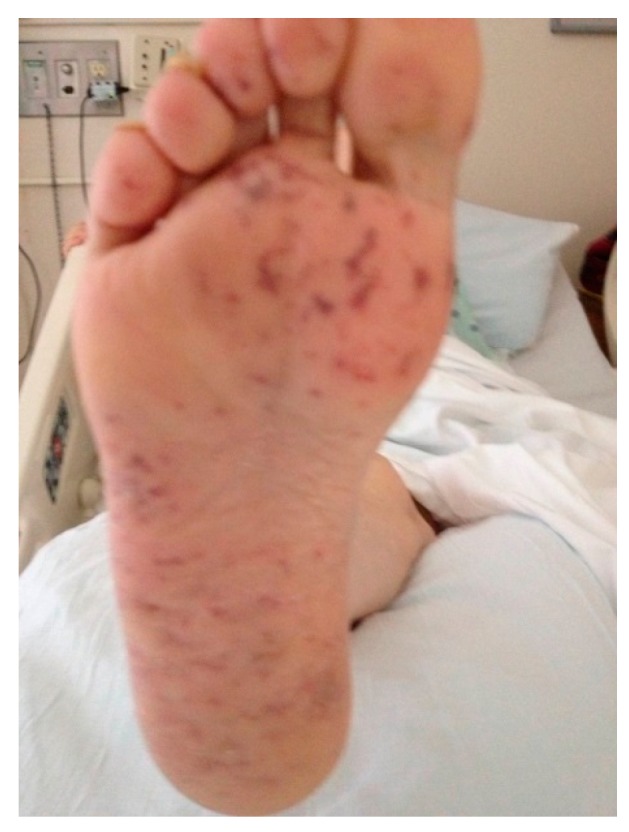
Photograph of painful livedo reticularis. Painful livedo reticularis on the sole of the right foot of a 57-year-old woman with diabetes, coronary artery disease, hyperlipidemia, stage V chronic kidney disease who underwent right carotid artery stenting for amaurosis fugax. The procedure was done by interventional radiology through a right femoral artery approach. The patient was placed on clopidogrel after stenting. Serum creatinine was 4.86 mg/dL on admission for the carotid artery stenting but dropped to baseline 3.74 mg/dL after the procedure. She presented a week later complaining of foot pain and was found to have tender petechial spots on the sole of her right foot. Creatinine was now 4.66 mg/dL, but with supportive care came back to baseline at 3.64 mg/dL on discharge. The picture is used with the patient’s permission.

The syndrome of CCE has various clinical features (Table 1). The most common extra-renal manifestation of CCE is in skin, with reported frequency of 75% to 96%. Skin manifestations, usually limited to the lower extremities, are most commonly livedo reticularis (purplish rash) and blue toe syndrome, ulceration, gangrene, purpura, small nail bed infarcts, leg, foot, or toe pain [41,46,47]. Skin findings frequently occur on the trunk and rarely on the upper extremities, but we diagnosed one case of painful livedo reticularis on the sole of the right foot (Figure 2).

Other organs, including gastrointestinal tract, retina, central nervous system, skeletal muscle, pancreas, liver, spinal cord, prostate, adrenal glands, and heart may also be affected [2]. The distribution of end-organ damage depends on the anatomic location of the original atherosclerotic emboli and the extent of organ involvement. Visceral ischemia presents as abdominal pain and flank or back pain, gastrointestinal bleeding, infarction and obstruction (caused by gastrointestinal ischemia), diarrhea, cholecystitis, and splenic infarcts. Pancreatic ischemia leads to acute pancreatitis with elevated pancreatic enzymes. The retina is also a frequent target organ of atheroembolism, with the incidence varying from 11% to 25% [48]. Emboli in retinal arterioles can cause sudden blindness, amaurosis fugax, and be seen on fundoscopy as bright yellow retinal plaques (Hollenhorst plaques). Neurological features include headache, amaurosis fugax, stroke, altered mental status, paraparesis, mononeuropathy, cerebral and spinal cord infarction [49]. The musculoskeletal features include muscle pain, arthralgias, and rhabdomyolysis.

Constitutional signs and symptoms may include fever, anorexia, fatigue, weight loss, malaise, and myalgias. These may be only non-specific inflammatory responses with increased erythrocyte sedimentation rate and eosinophilia. Hypocomplementemia can also be observed in AERD, because the CCs from atheromatous plaques can activate the complement pathway [50].

## 7. Treatments

There is no specific therapy for AERD. Withdrawal of any form of anticoagulants, postponing aortic catheterization and surgery should be considered firstly to avoid CCE recurrence. The aim of treatment is to prevent the progression of tissue ischemia and further showering of cholesterol crystals or provide supportive care in the event of renal failure.

### 7.1. Corticosteroids

The aim of corticosteroid use is to reduce the reactive inflammatory response along with atheroembolization, but the effects of steroids remain controversial. Some studies demonstrated that oral prednisolone administered at a dose of 1 mg/kg/day led to overall clinical improvement and improved renal outcomes in CCE patients [51]. Fabbian et al. reported a case of CCE treated successfully with high-dose methylprednisolone (250 mg intravenously) followed by oral administration of prednisone 50 mg a day gradually tapered off over 6 months. [52]. A small case series of renal CCE patients [53] and individual case reports also demonstrated that corticosteroid treatment led to improvement in renal function and other clinical symptoms [47,51]. However, other studies showed that corticosteroids did not have a favorable effect on long-term renal outcomes and were even associated with an increased risk of mortality [54,55,56].

### 7.2. Lipid-Lowering Therapies

Statins may have a beneficial effect in AERD by contributing to plaque stabilization and regression through their lipid-lowering and anti-inflammatory properties [48,57]. Unfortunately, there has been no randomized trial of statin therapy in patients with severe aortic plaque [56]. A recent study reported that low-density lipoprotein apheresis (LDL-A) decreased the risk of maintenance dialysis in 49 CCE patients with renal dysfunction after 24 weeks [58]. Lipoprotein apheresis (LA) can significantly lower lipoprotein cholesterol levels, and is recommended in some countries in very high-risk patients with early or progressive cardiovascular disease (CVD) [59]. LDL-A may have beneficial effect on renal outcome of CCE.

### 7.3. Dialysis and Other Therapies

Patients with acute kidney injury may require dialysis therapy. Both peritoneal dialysis and hemodialysis have been shown to be adequate means of managing renal failure in such patients. Belenfant et al. recommend heparin-free dialysis to avoid anticoagulation and thereby prevent further embolization [60]. Surgical measures, such as endarterectomy, vessel ligation or bypass, may decrease the probability of further embolism [61]. There is no evidence that anti-inflammatory treatments and antiplatelet agents are beneficial.

## 8. Outcome

Cholesterol crystal microemboli are commonly associated with irreversible organ damage and a generally poor prognosis. Renal outcomes are variable, with some patients requiring maintenance dialysis and others showing improvement in renal function but with varying degrees of residual chronic kidney disease. About 30–55% of acute/subacute CCE patients need dialysis [7,62]. Renal function recovery has been reported in only 21–28% of such cases [62]. Another 23–32% of AERD patients progress to end-stage renal disease (ESRD) [48]. Preexisting chronic kidney disease is associated with increased risk of progressing to ESRD in AERD [48,62]. A prospective study of 25 patients using endovascular stent graft coverage of embolizing aortic lesions showed that 12% had stage IV chronic kidney disease, and two of them became dialysis-dependent. Although the patients had chronic renal insufficiency and received a contrast load with computerized tomography (CT) imaging, their kidney function did not worsen after endovascular treatment [63]. One and two-year patient survival rates of 87% and 75%, respectively, have been reported while the 4-year survival rate drops to 52% [60]. Mortality is high, varying from 64% to 81% [64,65,66]. The major cause of death is cardiovascular disease [67]. Age, diabetes, history of heart failure, baseline renal function, time course of decline in renal function, and extra-renal manifestations are risk factors for both ESRD and death [7].

## 9. Prevention

Prevention is important for AERD in large part because no effective treatment is available at present. Prophylaxis against further episodes of cholesterol embolization is beneficial. CCE is a serious complication of invasive vascular procedures, with the incidence of CCE ranging from 1.4% to 2.0% [14,68]. Some experts recommend limiting excess anticoagulation and restricting angiography and surgical procedures as much as possible in patients with severe atherosclerosis [60,69]. New noninvasive diagnostic tools, such as spiral computed tomographic angiography, angiomagnetic resonance, and duplex ultrasonography, might reduce catheter-induced CCE. Technical considerations such as minimizing contrast angiography during invasive procedures, use of intravascular ultrasound or transesophageal echocardiography, and minimizing wire/catheter manipulation and balloon angioplasty all play important roles in minimizing kidney injury and morbidity related to further embolic phenomena [63,70].

## 10. Conclusions

The expansion of intravascular catheterization to treat a wide variety of atherosclerotic diseases has greatly reduced mortality from cardiovascular diseases in the last decade but has also increased the incidence of CCE, a complex phenomenon with its own significant morbidity and mortality. However, diagnosis of CCE remains a clinical challenge since its symptoms are non-specific symptoms. Understanding the risk factors for and causes of CCE should help physicians diagnose the syndrome in patients with a history of atherosclerosis and an invasive arterial procedure who present with acute/subacute or chronic renal failure. Awareness of those risk factors and causes may also help physicians reduce damage from intravascular intervention by considering non-invasive alternatives or preparing for it when an invasive intravascular procedure cannot be avoided.

## Figures and Tables

**Table 1:** The clinical presentations of cholesterol crystal embolization.

Organ	Clinical Presentations
**Kidney**	Acute or subacute kidney injury
	Renal infarction
	Chronic kidney disease
	Renal allograft failure
	Severe hard-to-control hypertension
	Extra-renal organs
**Skin**	Livedo reticularis
	Blue toe syndrome
	Ulceration and gangrene
	Purpura
	Small nail bed infarcts
	Leg, foot, or toe pain
**Gastrointestinal System**	Abdominal, flank, or back pain
	Gastrointestinal bleeding
	Diarrhea
	Bowel ischemia, infarction, and obstruction
	Pancreatitis, cholecystitis, and abnormal liver tests
	Splenic infarcts
**Eyes**	Amaurosis fugax
	Sudden blindness
	Retinal plaques (Hollenhorst plaques)
**Central Nervous System**	Headache
	Amaurosis fugax
	Stroke
	Transient ischaemic attacks
	Altered mental status
	Paraparesis
	Mononeuropathy
	Cerebral infarction
	Spinal cord infarction
**Muscle**	Muscle pain
	Arthralgias
	Rhabdomyolysis
	Systemic signs
	Fever
	Anorexia
	Fatigue
	Weight loss
	Malaise
	Myalgia
